# 2-(5,6-Dihydro­benzimidazo[1,2-*c*]quinazolin-6-yl)-5-meth­oxy­phenol

**DOI:** 10.1107/S1600536811034027

**Published:** 2011-08-27

**Authors:** Naser Eltaher Eltayeb, Siang Guan Teoh, Suchada Chantrapromma, Hoong-Kun Fun

**Affiliations:** a School of Chemical Sciences, Universiti Sains Malaysia, 11800 USM, Penang, Malaysia; b Department of Chemistry, Faculty of Pure and Applied Sciences, International University of Africa, Sudan; c Crystal Materials Research Unit, Department of Chemistry, Faculty of Science, Prince of Songkla University, Hat-Yai, Songkhla 90112, Thailand; d X-ray Crystallography Unit, School of Physics, Universiti Sains Malaysia, 11800 USM, Penang, Malaysia

## Abstract

In the title quinazoline derivative, C_21_H_17_N_3_O_2_, the benzimidazole unit makes dihedral angles of 8.29 (5) and 81.79 (5)° with the benzene rings of the quinazoline and meth­oxy­phenol units, respectively. The nitro­gen-containing six-membered ring adopts a half-chair conformation. In the crystal, the mol­ecules are linked through O—H⋯N hydrogen bonds into screw chains along the *b* axis; adjacent chains are further connected by N—H⋯O hydrogen bonds, thereby forming a two-dimensional network lying parallel to the *bc* plane. Weak C—H⋯π and π⋯π inter­actions with centroid–centroid distances of 3.5258 (8) and 3.7184 (7) Å are present and N⋯O [2.6816 (15) and 3.0519 (15) Å] short contacts also occur.

## Related literature

For background to benzoheterocyclic derivatives and their applications, see: Arienzo *et al.* (2007 ▶); Chassaing *et al.* (2008 ▶); Galarcei *et al.* (2008 ▶); Kumar & Rajput (2009 ▶); Kung *et al.* (2009 ▶); Podunavac-Kuzmanovic & Cvetkovic (2010 ▶); Via *et al.* (2001 ▶); Xue *et al.* (2011 ▶); Zhang *et al.* (2009 ▶). For related structures, see: Eltayeb *et al.* (2007 ▶, 2009 ▶, 2011*a* ▶,*b* ▶). For reference bond-length data, see: Allen *et al.* (1987 ▶). For ring conformations, see: Cremer & Pople (1975 ▶). For the stability of the temperature controller used in the data collection, see: Cosier & Glazer (1986 ▶).
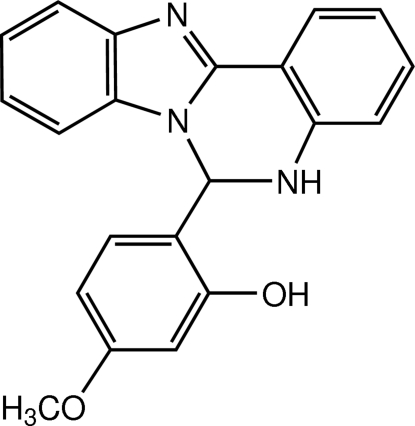


## Experimental

### 

#### Crystal data

C_21_H_17_N_3_O_2_
*M* *_r_* = 343.38
Monoclinic, 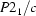

*a* = 9.5408 (1) Å
*b* = 15.6503 (2) Å
*c* = 11.7609 (1) Å
β = 110.408 (1)°
*V* = 1645.87 (3) Å^3^
*Z* = 4
Mo *K*α radiation
μ = 0.09 mm^−1^
*T* = 100 K
0.28 × 0.25 × 0.22 mm

#### Data collection

Bruker APEXII CCD diffractometer
Absorption correction: multi-scan (*SADABS*; Bruker, 2005 ▶) *T*_min_ = 0.974, *T*_max_ = 0.980
31713 measured reflections
6610 independent reflections
4637 reflections with *I* > 2σ(*I*)
*R*_int_ = 0.039

#### Refinement

*R*[*F* ^2^ > 2σ(*F* ^2^)] = 0.059
*wR*(*F* ^2^) = 0.139
*S* = 1.05
6610 reflections
244 parameters
H atoms treated by a mixture of independent and constrained refinement
Δρ_max_ = 0.43 e Å^−3^
Δρ_min_ = −0.30 e Å^−3^

### 

Data collection: *APEX2* (Bruker, 2005 ▶); cell refinement: *SAINT* (Bruker, 2005 ▶); data reduction: *SAINT*; program(s) used to solve structure: *SHELXTL* (Sheldrick, 2008 ▶); program(s) used to refine structure: *SHELXTL*; molecular graphics: *SHELXTL*; software used to prepare material for publication: *SHELXTL* and *PLATON* (Spek, 2009 ▶).

## Supplementary Material

Crystal structure: contains datablock(s) global, I. DOI: 10.1107/S1600536811034027/hb6371sup1.cif

Structure factors: contains datablock(s) I. DOI: 10.1107/S1600536811034027/hb6371Isup2.hkl

Supplementary material file. DOI: 10.1107/S1600536811034027/hb6371Isup3.cml

Additional supplementary materials:  crystallographic information; 3D view; checkCIF report

## Figures and Tables

**Table 1:** Hydrogen-bond geometry (Å, °) *Cg*4 is the centroid of the C15–C20 ring.

*D*—H⋯*A*	*D*—H	H⋯*A*	*D*⋯*A*	*D*—H⋯*A*
N3—H1*N*3⋯O2^i^	0.907 (19)	2.211 (19)	3.0519 (15)	153.8 (17)
O1—H1*O*1⋯N2^ii^	0.98 (2)	1.72 (2)	2.6816 (15)	168 (2)
C2—H2*A*⋯*Cg*4	0.95	2.85	3.6277 (16)	140

Symmetry codes: (i) 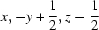; (ii) 
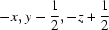
.

**Fig. 1.**
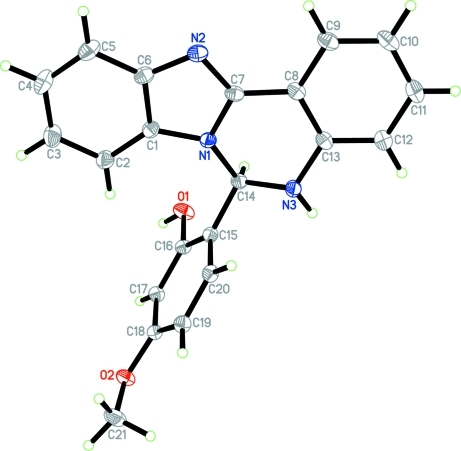
The molecular structure of the title compound, with 50% probability displacement ellipsoids.

**Fig. 2.**
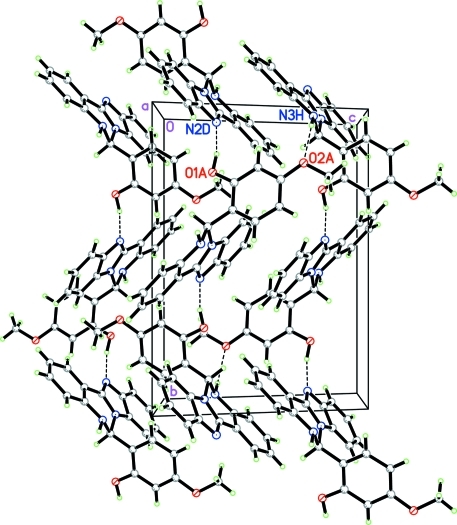
The crystal packing of the title compound viewed down the a axis, showing 2D network parallel to the bc plane. Hydrogen bonds are shown as dashed lines.

## supplementary crystallographic information

### Comment

Benzoheterocyclic derivatives have been used widely in the pharmaceutical industry and medicine due to their diverse pharmaceutical activities (Arienzo *et al.*, 2007; Chassaing *et al.*, 2008; Kumar *et al.*, 2009; Kung *et al.*, 2009; Podunavac-Kuzmanovic & Cvetkovic, 2010; Zhang *et al.*, 2009) including inhibition against enteroviruses (Xue *et al.*, 2011) and potent antitumor activity (Galarcei *et al.*, 2008; Via *et al.*, 2001). Due to their interesting activities, the benzimidazole and quinazoline scaffolds were selected for our ongoing structural studies (Eltayeb *et al.*, 2007; 2009; 2011*a*; 2011*b*).

In the title compound (I) (Fig. 1), the benzimidazole ring system (C1–C7/N1–N2) is planar with the *r.m.s*. of 0.0086 (1) Å with the most deviation for atom C1 of 0.0183 (1) Å. The benzimidazole makes the dihedral angle of 8.29 (5)° with the C8–C13 benzene ring of the quinazoline moiety (C7–C14/N1/N3). The nitrogen six-membered ring adopts a half-chair conformation with the puckering parameter Q = 0.3941 (13) Å, θ = 59.34 (19)° and φ = 277.7 (2)° (Cremer & Pople, 1975). The orientation of the 5-methoxyphenol can be indicated by the dihedral angle between the phenol ring and benzimidazole of 81.79 (5)°. The methoxy substituted is slightly twisted from its attached benzene ring with the torsion angle C21–O2–C18–C19 = 8.93 (17)°. The bond lengths agree with the literature values (Allen *et al.*, 1987).

In the crystal structure of (I) as shown Fig. 2, the molecules are linked through O—H···N hydrogen bonds (Table 1) into screw chains along the *b* axis. The adjacent screw chains are further connected by N—H···O hydrogen bonds (Table 1) forming the two-dimensional network parallel to the *bc* plane. The crystal is further stabilized by C—H···π weak interactions (Table 1). π···π interactions were also observed with centroid···centroid distances: *Cg*_1_···*Cg*_3_^iii^ = 3.7184 (7) Å and *Cg*_2_···*Cg*_2_^iv^ = 3.5258 (8) Å; *Cg*_1_, *Cg*_2_ and *Cg*_3_ are the centroids of C1/C6/C7/N1–N2, C1–C6 and C8–C13 rings, respectively (symmetry codes: (iii) = -x, 1-y, -z and (iv) = -x, 1-y 1-z). N···O[2.6816 (15) and 3.0519 (15) Å] short contacts were also observed.

### Experimental

The title compound was synthesized by adding 2-hydroxy-4-methoxybenzaldehyde (0.304 g, 2.0 mmol) to a solution of 2-(2-aminophenyl)-1*H*-benzimidazole (0.418 g, 2.0 mmol) in ethanol (30 mL). The mixture was refluxed with stirring for 2 hrs. The color of the resulting solution was pale-yellow. Pale-yellow blocks were formed after three weeks of slow evaporation of ethanol at room temperature.

### Refinement

H atom attached to O1 and N3 were located in a difference maps and refined isotropically. The remaining H atoms were positioned geometrically and allowed to ride on their parent atoms, with d(C-H) = 0.95 Å for aromatic and CH; and 0.98 Å for CH_3_. The *U*_iso_ values were constrained to be 1.5*U*_eq_ of the carrier atom for methyl H atoms and 1.2*U*_eq_ for the remaining H atoms. A rotating group model was used for the methyl groups. The highest residual electron density peak is located at 0.67 Å from C13 and the deepest hole is located at 0.45 Å from C14.

### Crystal data

**Table:**

C_21_H_17_N_3_O_2_	*F*(000) = 720
*M**_r_* = 343.38	*D*_x_ = 1.386 Mg m^−^^3^
Monoclinic, *P*2_1_/*c*	Mo *K*α radiation, λ = 0.71073 Å
Hall symbol: -P 2ybc	Cell parameters from 6610 reflections
*a* = 9.5408 (1) Å	θ = 2.3–33.8°
*b* = 15.6503 (2) Å	µ = 0.09 mm^−^^1^
*c* = 11.7609 (1) Å	*T* = 100 K
β = 110.408 (1)°	Block, pale yellow
*V* = 1645.87 (3) Å^3^	0.28 × 0.25 × 0.22 mm
*Z* = 4	

### Data collection

**Table:**

Bruker APEXII CCD diffractometer	6610 independent reflections
Radiation source: sealed tube	4637 reflections with *I* > 2σ(*I*)
graphite	*R*_int_ = 0.039
φ and ω scans	θ_max_ = 33.8°, θ_min_ = 2.3°
Absorption correction: multi-scan (*SADABS*; Bruker, 2005)	*h* = −14→14
*T*_min_ = 0.974, *T*_max_ = 0.980	*k* = −24→22
31713 measured reflections	*l* = −18→18

### Refinement

**Table:**

Refinement on *F*^2^	Primary atom site location: structure-invariant direct methods
Least-squares matrix: full	Secondary atom site location: difference Fourier map
*R*[*F*^2^ > 2σ(*F*^2^)] = 0.059	Hydrogen site location: inferred from neighbouring sites
*wR*(*F*^2^) = 0.139	H atoms treated by a mixture of independent and constrained refinement
*S* = 1.05	*w* = 1/[σ^2^(*F*_o_^2^) + (0.0535*P*)^2^ + 0.6883*P*] where *P* = (*F*_o_^2^ + 2*F*_c_^2^)/3
6610 reflections	(Δ/σ)_max_ = 0.001
244 parameters	Δρ_max_ = 0.43 e Å^−^^3^
0 restraints	Δρ_min_ = −0.30 e Å^−^^3^

### Special details

**Table:**

Experimental. The crystal was placed in the cold stream of an Oxford Cryosystems Cobra open-flow nitrogen cryostat (Cosier & Glazer, 1986) operating at 120.0 (1) K.
Geometry. All esds (except the esd in the dihedral angle between two l.s. planes) are estimated using the full covariance matrix. The cell esds are taken into account individually in the estimation of esds in distances, angles and torsion angles; correlations between esds in cell parameters are only used when they are defined by crystal symmetry. An approximate (isotropic) treatment of cell esds is used for estimating esds involving l.s. planes.
Refinement. Refinement of F^2^ against ALL reflections. The weighted R-factor wR and goodness of fit S are based on F^2^, conventional R-factors R are based on F, with F set to zero for negative F^2^. The threshold expression of F^2^ > 2sigma(F^2^) is used only for calculating R-factors(gt) etc. and is not relevant to the choice of reflections for refinement. R-factors based on F^2^ are statistically about twice as large as those based on F, and R- factors based on ALL data will be even larger.

### Fractional atomic coordinates and isotropic or equivalent isotropic displacement parameters (Å^2^)

**Table:**

	*x*	*y*	*z*	*U*_iso_*/*U*_eq_	
O1	0.14755 (11)	0.22525 (6)	0.27396 (8)	0.0225 (2)	
O2	0.48793 (10)	0.20256 (6)	0.67860 (8)	0.01967 (19)	
N1	0.09967 (11)	0.45602 (7)	0.26995 (9)	0.0155 (2)	
N2	−0.06837 (12)	0.56241 (7)	0.21091 (10)	0.0195 (2)	
N3	0.32315 (12)	0.45264 (7)	0.22680 (10)	0.0181 (2)	
C1	−0.00334 (14)	0.44114 (8)	0.32723 (11)	0.0169 (2)	
C2	−0.01453 (15)	0.37959 (9)	0.40964 (12)	0.0214 (3)	
H2A	0.0559	0.3344	0.4361	0.026*	
C3	−0.13327 (16)	0.38755 (9)	0.45104 (13)	0.0261 (3)	
H3A	−0.1438	0.3471	0.5078	0.031*	
C4	−0.23825 (16)	0.45360 (10)	0.41148 (14)	0.0276 (3)	
H4A	−0.3193	0.4562	0.4407	0.033*	
C5	−0.22662 (15)	0.51499 (9)	0.33108 (13)	0.0251 (3)	
H5A	−0.2975	0.5600	0.3051	0.030*	
C6	−0.10683 (14)	0.50852 (8)	0.28924 (11)	0.0185 (2)	
C7	0.05266 (14)	0.52788 (8)	0.19987 (11)	0.0165 (2)	
C8	0.13443 (14)	0.55651 (8)	0.12348 (11)	0.0170 (2)	
C9	0.08353 (15)	0.62216 (8)	0.03824 (11)	0.0206 (3)	
H9A	−0.0070	0.6510	0.0303	0.025*	
C10	0.16415 (16)	0.64543 (9)	−0.03470 (11)	0.0230 (3)	
H10A	0.1282	0.6894	−0.0935	0.028*	
C11	0.29812 (16)	0.60404 (9)	−0.02133 (11)	0.0233 (3)	
H11A	0.3534	0.6199	−0.0713	0.028*	
C12	0.35181 (16)	0.53980 (9)	0.06425 (11)	0.0209 (3)	
H12A	0.4444	0.5128	0.0737	0.025*	
C13	0.26941 (14)	0.51475 (8)	0.13664 (11)	0.0171 (2)	
C14	0.21403 (13)	0.39827 (8)	0.25378 (10)	0.0157 (2)	
H14A	0.1659	0.3593	0.1836	0.019*	
C15	0.29119 (13)	0.34584 (8)	0.36554 (10)	0.0148 (2)	
C16	0.25404 (13)	0.25964 (8)	0.37158 (11)	0.0159 (2)	
C17	0.32374 (13)	0.21322 (8)	0.47774 (10)	0.0162 (2)	
H17A	0.2994	0.1547	0.4821	0.019*	
C18	0.42921 (13)	0.25281 (8)	0.57752 (10)	0.0154 (2)	
C19	0.46985 (14)	0.33780 (8)	0.57194 (11)	0.0171 (2)	
H19A	0.5438	0.3643	0.6389	0.021*	
C20	0.39884 (13)	0.38268 (8)	0.46523 (11)	0.0163 (2)	
H20A	0.4251	0.4408	0.4605	0.020*	
C21	0.57945 (16)	0.24524 (10)	0.78736 (12)	0.0258 (3)	
H21A	0.6014	0.2060	0.8563	0.039*	
H21B	0.5261	0.2953	0.8016	0.039*	
H21C	0.6733	0.2635	0.7783	0.039*	
H1N3	0.3958 (19)	0.4194 (12)	0.2166 (15)	0.028 (4)*	
H1O1	0.126 (2)	0.1665 (15)	0.291 (2)	0.059 (6)*	

### Atomic displacement parameters (Å^2^)

**Table:**

	*U*^11^	*U*^22^	*U*^33^	*U*^12^	*U*^13^	*U*^23^
O1	0.0248 (5)	0.0166 (5)	0.0191 (4)	−0.0058 (4)	−0.0014 (4)	0.0006 (3)
O2	0.0217 (4)	0.0185 (4)	0.0154 (4)	−0.0019 (4)	0.0021 (3)	0.0027 (3)
N1	0.0152 (5)	0.0139 (5)	0.0175 (4)	0.0018 (4)	0.0059 (4)	0.0019 (4)
N2	0.0190 (5)	0.0167 (5)	0.0214 (5)	0.0031 (4)	0.0054 (4)	0.0003 (4)
N3	0.0185 (5)	0.0166 (5)	0.0214 (5)	0.0033 (4)	0.0098 (4)	0.0042 (4)
C1	0.0155 (5)	0.0172 (6)	0.0180 (5)	−0.0007 (4)	0.0057 (4)	−0.0019 (4)
C2	0.0211 (6)	0.0196 (6)	0.0253 (6)	0.0019 (5)	0.0103 (5)	0.0025 (5)
C3	0.0277 (7)	0.0241 (7)	0.0316 (7)	−0.0024 (6)	0.0166 (6)	0.0014 (5)
C4	0.0239 (7)	0.0271 (7)	0.0378 (8)	−0.0020 (6)	0.0184 (6)	−0.0052 (6)
C5	0.0201 (6)	0.0220 (7)	0.0345 (7)	0.0032 (5)	0.0113 (5)	−0.0031 (5)
C6	0.0168 (6)	0.0164 (6)	0.0214 (5)	0.0010 (5)	0.0054 (4)	−0.0025 (4)
C7	0.0175 (5)	0.0130 (5)	0.0165 (5)	0.0007 (4)	0.0028 (4)	−0.0004 (4)
C8	0.0203 (6)	0.0133 (5)	0.0160 (5)	−0.0011 (4)	0.0048 (4)	−0.0005 (4)
C9	0.0243 (6)	0.0156 (6)	0.0185 (5)	−0.0002 (5)	0.0031 (5)	0.0011 (4)
C10	0.0311 (7)	0.0169 (6)	0.0169 (5)	−0.0034 (5)	0.0034 (5)	0.0021 (4)
C11	0.0313 (7)	0.0207 (6)	0.0181 (5)	−0.0069 (5)	0.0091 (5)	−0.0005 (5)
C12	0.0244 (6)	0.0194 (6)	0.0201 (6)	−0.0012 (5)	0.0094 (5)	−0.0002 (5)
C13	0.0215 (6)	0.0134 (5)	0.0160 (5)	−0.0015 (4)	0.0061 (4)	−0.0002 (4)
C14	0.0170 (5)	0.0134 (5)	0.0170 (5)	0.0017 (4)	0.0063 (4)	0.0006 (4)
C15	0.0150 (5)	0.0127 (5)	0.0173 (5)	0.0009 (4)	0.0064 (4)	0.0009 (4)
C16	0.0144 (5)	0.0154 (5)	0.0166 (5)	0.0001 (4)	0.0039 (4)	−0.0002 (4)
C17	0.0173 (5)	0.0126 (5)	0.0181 (5)	−0.0005 (4)	0.0056 (4)	0.0010 (4)
C18	0.0145 (5)	0.0162 (6)	0.0155 (5)	0.0014 (4)	0.0053 (4)	0.0013 (4)
C19	0.0169 (5)	0.0164 (6)	0.0173 (5)	−0.0027 (4)	0.0049 (4)	−0.0024 (4)
C20	0.0171 (5)	0.0124 (5)	0.0202 (5)	−0.0012 (4)	0.0077 (4)	−0.0003 (4)
C21	0.0268 (7)	0.0277 (7)	0.0165 (5)	−0.0066 (6)	−0.0005 (5)	0.0030 (5)

### Geometric parameters (Å, °)

**Table:**

O1—C16	1.3516 (14)	C8—C13	1.4038 (18)
O1—H1O1	0.98 (2)	C9—C10	1.386 (2)
O2—C18	1.3716 (14)	C9—H9A	0.9500
O2—C21	1.4373 (15)	C10—C11	1.392 (2)
N1—C7	1.3739 (15)	C10—H10A	0.9500
N1—C1	1.3913 (16)	C11—C12	1.3880 (18)
N1—C14	1.4793 (15)	C11—H11A	0.9500
N2—C7	1.3217 (17)	C12—C13	1.4012 (18)
N2—C6	1.3894 (17)	C12—H12A	0.9500
N3—C13	1.3974 (16)	C14—C15	1.5060 (16)
N3—C14	1.4626 (16)	C14—H14A	1.0000
N3—H1N3	0.908 (18)	C15—C20	1.3863 (16)
C1—C2	1.3967 (18)	C15—C16	1.4030 (17)
C1—C6	1.4067 (18)	C16—C17	1.3964 (16)
C2—C3	1.3859 (19)	C17—C18	1.3964 (17)
C2—H2A	0.9500	C17—H17A	0.9500
C3—C4	1.401 (2)	C18—C19	1.3934 (17)
C3—H3A	0.9500	C19—C20	1.3909 (17)
C4—C5	1.379 (2)	C19—H19A	0.9500
C4—H4A	0.9500	C20—H20A	0.9500
C5—C6	1.3967 (19)	C21—H21A	0.9800
C5—H5A	0.9500	C21—H21B	0.9800
C7—C8	1.4509 (18)	C21—H21C	0.9800
C8—C9	1.3989 (17)		
			
C16—O1—H1O1	110.2 (13)	C12—C11—C10	120.74 (13)
C18—O2—C21	116.28 (10)	C12—C11—H11A	119.6
C7—N1—C1	106.83 (10)	C10—C11—H11A	119.6
C7—N1—C14	121.61 (10)	C11—C12—C13	119.99 (13)
C1—N1—C14	129.58 (10)	C11—C12—H12A	120.0
C7—N2—C6	105.00 (10)	C13—C12—H12A	120.0
C13—N3—C14	117.95 (10)	N3—C13—C12	121.37 (12)
C13—N3—H1N3	113.2 (11)	N3—C13—C8	119.16 (11)
C14—N3—H1N3	109.2 (11)	C12—C13—C8	119.36 (11)
N1—C1—C2	133.57 (12)	N3—C14—N1	106.57 (10)
N1—C1—C6	104.88 (11)	N3—C14—C15	109.75 (10)
C2—C1—C6	121.49 (12)	N1—C14—C15	112.25 (9)
C3—C2—C1	116.84 (12)	N3—C14—H14A	109.4
C3—C2—H2A	121.6	N1—C14—H14A	109.4
C1—C2—H2A	121.6	C15—C14—H14A	109.4
C2—C3—C4	121.79 (13)	C20—C15—C16	118.87 (11)
C2—C3—H3A	119.1	C20—C15—C14	120.27 (11)
C4—C3—H3A	119.1	C16—C15—C14	120.85 (10)
C5—C4—C3	121.50 (13)	O1—C16—C17	122.33 (11)
C5—C4—H4A	119.3	O1—C16—C15	117.93 (11)
C3—C4—H4A	119.3	C17—C16—C15	119.71 (11)
C4—C5—C6	117.53 (13)	C16—C17—C18	119.96 (11)
C4—C5—H5A	121.2	C16—C17—H17A	120.0
C6—C5—H5A	121.2	C18—C17—H17A	120.0
N2—C6—C5	128.86 (12)	O2—C18—C19	123.50 (11)
N2—C6—C1	110.31 (11)	O2—C18—C17	115.52 (11)
C5—C6—C1	120.83 (12)	C19—C18—C17	120.98 (11)
N2—C7—N1	112.91 (11)	C20—C19—C18	117.94 (11)
N2—C7—C8	127.69 (11)	C20—C19—H19A	121.0
N1—C7—C8	119.39 (11)	C18—C19—H19A	121.0
C9—C8—C13	119.79 (12)	C15—C20—C19	122.49 (11)
C9—C8—C7	122.91 (12)	C15—C20—H20A	118.8
C13—C8—C7	117.30 (11)	C19—C20—H20A	118.8
C10—C9—C8	120.52 (13)	O2—C21—H21A	109.5
C10—C9—H9A	119.7	O2—C21—H21B	109.5
C8—C9—H9A	119.7	H21A—C21—H21B	109.5
C9—C10—C11	119.58 (12)	O2—C21—H21C	109.5
C9—C10—H10A	120.2	H21A—C21—H21C	109.5
C11—C10—H10A	120.2	H21B—C21—H21C	109.5
			
C7—N1—C1—C2	−178.82 (14)	C14—N3—C13—C8	35.42 (16)
C14—N1—C1—C2	17.3 (2)	C11—C12—C13—N3	−177.47 (12)
C7—N1—C1—C6	−1.59 (13)	C11—C12—C13—C8	−1.42 (19)
C14—N1—C1—C6	−165.47 (11)	C9—C8—C13—N3	176.42 (11)
N1—C1—C2—C3	177.56 (13)	C7—C8—C13—N3	−4.28 (17)
C6—C1—C2—C3	0.70 (19)	C9—C8—C13—C12	0.28 (18)
C1—C2—C3—C4	0.6 (2)	C7—C8—C13—C12	179.57 (11)
C2—C3—C4—C5	−1.3 (2)	C13—N3—C14—N1	−49.22 (13)
C3—C4—C5—C6	0.7 (2)	C13—N3—C14—C15	−171.00 (10)
C7—N2—C6—C5	−179.60 (13)	C7—N1—C14—N3	37.49 (14)
C7—N2—C6—C1	1.18 (14)	C1—N1—C14—N3	−160.71 (11)
C4—C5—C6—N2	−178.49 (13)	C7—N1—C14—C15	157.67 (11)
C4—C5—C6—C1	0.6 (2)	C1—N1—C14—C15	−40.53 (16)
N1—C1—C6—N2	0.28 (14)	N3—C14—C15—C20	42.18 (15)
C2—C1—C6—N2	177.93 (11)	N1—C14—C15—C20	−76.13 (14)
N1—C1—C6—C5	−179.00 (11)	N3—C14—C15—C16	−138.89 (12)
C2—C1—C6—C5	−1.36 (19)	N1—C14—C15—C16	102.80 (13)
C6—N2—C7—N1	−2.29 (14)	C20—C15—C16—O1	179.23 (11)
C6—N2—C7—C8	176.73 (12)	C14—C15—C16—O1	0.28 (17)
C1—N1—C7—N2	2.53 (14)	C20—C15—C16—C17	1.05 (18)
C14—N1—C7—N2	167.97 (10)	C14—C15—C16—C17	−177.90 (11)
C1—N1—C7—C8	−176.58 (10)	O1—C16—C17—C18	−177.68 (11)
C14—N1—C7—C8	−11.14 (17)	C15—C16—C17—C18	0.42 (18)
N2—C7—C8—C9	−7.4 (2)	C21—O2—C18—C19	8.93 (17)
N1—C7—C8—C9	171.60 (11)	C21—O2—C18—C17	−171.12 (11)
N2—C7—C8—C13	173.36 (12)	C16—C17—C18—O2	178.11 (11)
N1—C7—C8—C13	−7.68 (17)	C16—C17—C18—C19	−1.94 (18)
C13—C8—C9—C10	1.00 (19)	O2—C18—C19—C20	−178.13 (11)
C7—C8—C9—C10	−178.26 (12)	C17—C18—C19—C20	1.92 (18)
C8—C9—C10—C11	−1.12 (19)	C16—C15—C20—C19	−1.06 (18)
C9—C10—C11—C12	0.0 (2)	C14—C15—C20—C19	177.89 (11)
C10—C11—C12—C13	1.3 (2)	C18—C19—C20—C15	−0.41 (18)
C14—N3—C13—C12	−148.52 (12)		

### Hydrogen-bond geometry (Å, °)

**Table:**

*Cg*4 is the centroid of the C15–C20 ring.

**Table:**

*D*—H···*A*	*D*—H	H···*A*	*D*···*A*	*D*—H···*A*
N3—H1N3···O2^i^	0.907 (19)	2.211 (19)	3.0519 (15)	153.8 (17)
O1—H1O1···N2^ii^	0.98 (2)	1.72 (2)	2.6816 (15)	168 (2)
C2—H2A···Cg4	0.95	2.85	3.6277 (16)	140

Symmetry codes: (i) *x*, −*y*+1/2, *z*−1/2; (ii) −*x*, *y*−1/2, −*z*+1/2.
